# SbCOMT (Bmr12) is involved in the biosynthesis of tricin-lignin in sorghum

**DOI:** 10.1371/journal.pone.0178160

**Published:** 2017-06-08

**Authors:** Aymerick Eudes, Tanmoy Dutta, Kai Deng, Nicolas Jacquet, Anagh Sinha, Veronica T. Benites, Edward E. K. Baidoo, Aurore Richel, Scott E. Sattler, Trent R. Northen, Seema Singh, Blake A. Simmons, Dominique Loqué

**Affiliations:** 1 Joint BioEnergy Institute, EmeryStation East, Emeryville, California, United States of America; 2 Environmental Genomics and Systems Biology Division, Lawrence Berkeley National Laboratory, Berkeley, California, United States of America; 3 Biomass Science and Conversion Technology Department, Sandia National Laboratories, Livermore, California, United States of America; 4 Biotechnology and Bioengineering Department, Sandia National Laboratories, Livermore, California, United States of America; 5 Laboratory of Biological and Industrial Chemistry, University of Liege, Gembloux Agro-Bio Tech, Gembloux, Belgium; 6 Department of Molecular and Cell Biology, University of California, Berkeley, California, United States of America; 7 Biological Systems and Engineering Division, Lawrence Berkeley National Laboratory, Berkeley, California, United States of America; 8 Wheat, Sorghum, and Forage Research Unit, USDA-ARS, Lincoln, Nebraska, United States of America; 9 Joint Genome Institute, Walnut Creek, California, United States of America; 10 Department of Plant and Microbial Biology, University of California, Berkeley, California, California, United States of America; 11 Université Lyon 1, INSA de Lyon, CNRS, UMR5240, Microbiologie, Adaptation et Pathogénie, Villeurbanne, France; Tallinn University of Technology, ESTONIA

## Abstract

Lignin in plant biomass represents a target for engineering strategies towards the development of a sustainable bioeconomy. In addition to the conventional lignin monomers, namely *p*-coumaryl, coniferyl and sinapyl alcohols, tricin has been shown to be part of the native lignin polymer in certain monocot species. Because tricin is considered to initiate the polymerization of lignin chains, elucidating its biosynthesis and mechanism of export to the cell wall constitute novel challenges for the engineering of bioenergy crops. Late steps of tricin biosynthesis require two methylation reactions involving the pathway intermediate selgin. It has recently been demonstrated in rice and maize that caffeate *O*-methyltransferase (COMT) involved in the synthesis syringyl (S) lignin units derived from sinapyl alcohol also participates in the synthesis of tricin *in planta*. In this work, we validate in sorghum (*Sorghum bicolor* L.) that the *O*-methyltransferase responsible for the production of S lignin units (SbCOMT / Bmr12) is also involved in the synthesis of lignin-linked tricin. In particular, we show that biomass from the sorghum *bmr12* mutant contains lower level of tricin incorporated into lignin, and that SbCOMT can methylate the tricin precursors luteolin and selgin. Our genetic and biochemical data point toward a general mechanism whereby COMT is involved in the synthesis of both tricin and S lignin units.

## Introduction

**Fig 1:**
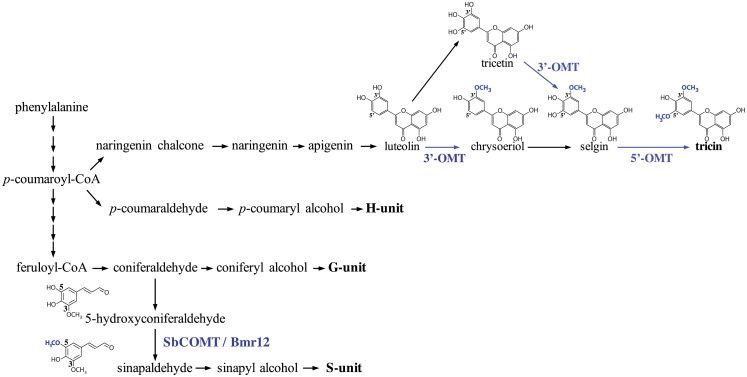
Simplified representation of the lignin and tricin biosynthetic pathways from phenylalanine. Abbreviations are: Bmr12, Brown midrib12; OMT, *O*-methyltransferase; SbCOMT, *Sorghum bicolor* caffeate *O*-methyltransferase.

Lignin is a rigid and hydrophobic cell-wall polymer that played a central role in the evolutionary conquest of land by vascular plants. Lignin in angiosperms arises from the oxidative polymerization of phenylpropanoid-derived *p*-coumaryl, coniferyl and sinapyl alcohols, which leads to the formation of H, G, and S lignin units, respectively [1]. During the biosynthesis of these lignin monomers (or monolignols), the formation of sinapyl alcohol requires the 5-*O*-methylation of 5-hydroxyconiferaldehyde catalyzed by caffeate *O*-methyltransferase (COMT, EC 2.1.1.68) (Fig 1) [2,3].

Besides the presence of canonical H, G and S units, lignin exhibits compositional plasticity, as exemplified by the occurrence of the flavone tricin found in the lignin of several monocot species and the dicot alfalfa (*Medicago sativa*) [4]. In particular, tricin has been shown to react with monolignols under radical coupling conditions and the corresponding tricin-oligolignol metabolites were identified in maize extracts [5,6]. As a result, tricin monomers are found even in the highest molecular weight fractions of lignin and act as nucleation sites for lignification [5,6]. The biosynthesis of flavones starts with *p*-coumaroyl-CoA as a precursor, and tricin biosynthesis is achieved via 5’-*O*-methylation of selgin, which derives from chrysoeriol and luteolin (Fig 1) [7,8]. As examples, the two *O*-methyltransferases, OsCOMT1 from rice and ZmCOMT (encoded at the *Bm3* locus) from maize, perform *in vitro* the 3’-*O*-methylation of luteolin to produce chrysoeriol [9–11]. Moreover, the affinity of OsCOMT1 and ZmCOMT toward selgin is also demonstrated by their capacity to form tricin via dual 3’/5’-*O*-methylation of tricetin (Fig 1) [8,11,12]. Consequently, rice seedlings of an *OsCOMT1* T-DNA insertion mutant show reduction of methanol-extractable tricin [8], and biomass from the maize *bm3* mutant has lower levels of lignin-linked tricin [13]. Finally, OsCOMT1 and ZmCOMT are also known to methylate 5-hydroxyconiferaldehyde and/or 5-hydroxyferulic acid, and transgenic rice and maize plants downregulated, respectively, for *OsCOMT1* and *ZmCOMT*, exhibit lower amount of S lignin units [11,13–15]. Overall, these observations suggest a general mechanism whereby COMT is involved in the synthesis of both tricin and S lignin units.

Tricin is produced and found in the lignin of sorghum [4], but none of the enzymes involved in the last steps of its biosynthesis have been characterized in this important multipurpose crop. The sorghum *brown midrib12* (*bmr12*) mutant shows a reduction of S units in lignin due to a premature stop codon in the gene encoding for the COMT (SbCOMT) that methylates 5-hydroxyconiferaldehyde (Fig 1) [16–18]. Our objective in this work was to gain insight into the biosynthesis of tricin in sorghum and to determine the possible role of SbCOMT in the methylation step(s) of the tricin biosynthetic pathway.

## Materials and methods

### Plant material

Biomass was harvested from field grown wild-type and *bmr12* sorghum plants with the panicles removed as previously described [19]. Plants were grown in at the University of Nebraska Field Laboratory, Ithaca, NE (coordinates 41.163182, -96.410486). This land was rented to USDA-ARS sorghum project from the University of Nebraska Agriculture Research and Development Center (http://ardc.unl.edu). No special permission was required. The land has been in cropping systems for over 50 years. The endangered or protected species also do not apply [19].

### Extraction of methanol-soluble metabolites

Ball-milled biomass from wild-type and *bmr12* plants (50 mg) was mixed with 1 ml of 80% (v/v) methanol-water and shaken at 1,400 rpm for 15 min at 70°C. The mixture was cleared by centrifugation for 5 min, at 20,000 x *g*. This step was repeated five times. Extracts were pooled and cleared one more time by centrifugation (5 min, 20,000 × *g*), mixed with 3 mL of analytical grade water and filtered using Amicon Ultra centrifugal filters (3,000 Da MW cutoff regenerated cellulose membrane; EMD Millipore, Billerica, MA). An aliquot of the filtered extracts (1.5 mL) was dried under vacuum, re-suspended with 1 N HCl, and incubated at 95°C for 3 h. The mixture was subjected to three ethyl acetate partitioning steps. Ethyl acetate fractions were pooled, dried in vacuo, and re-suspended in 50% (v/v) methanol-water (150 μL) prior to high-performance liquid chromatography (HPLC), electrospray ionization (ESI), and time-of-flight (TOF) mass spectrometry (MS) analysis.

### 2D ^13^C–^1^H heteronuclear single quantum coherence (HSQC) NMR spectroscopy

Extracted and ball-milled biomass was used for the purification of cellulolytic lignin as previously described [20]. The gels were formed using DMSO-d_6_/pyridine-d_5_ (4:1) and sonicated until homogenous in a Branson 2510 table-top cleaner (Branson Ultrasonic Corporation, Danbury, CT). The homogeneous solutions were transferred to NMR tubes. HSQC spectra were acquired at 25°C using a Bruker Avance-600 MHz instrument equipped with a 5 mm inverse-gradient ^1^H/^13^C cryoprobe using a hsqcetgpsisp2.2 pulse program (ns = 400, ds = 16, number of increments = 256, d_1_ = 1.0 s) [21]. Chemical shifts were referenced to the central DMSO peak (δ_C_/δ_H_ 39.5/2.5 ppm). Assignment of the HSQC spectra was described elsewhere [22–26]. A semi-quantitative analysis of the volume integrals of the HSQC correlation peaks was performed using Bruker’s Topspin 3.1 (Macintosh) processing software. A Guassian apodization in F_2_ (LB = -0.50, GB = 0.001) and squared cosine-bell in F_1_ (LB = -0.10, GB = 0.001) were applied prior to 2D Fourier transformation. For volume integration of lignin and tricin aromatic signals, C_2_–H_2_ correlation from guaiacyl units (G), magnetically equivalent C_2_–H_2_/C_6_–H_6_ correlation from syringyl units (S), magnetically equivalent C_2’_–H_2’_/C_6’_–H_6’_ correlation from tricin units (T), and C_2_–H_2_ correlation from 5-hydroxyguaiacyl units (5OH-G) were used. S and T integrals were halved and the relative amounts of each are expressed as a fraction of the total.

### Cloning of SbCOMT

A cDNA solution from sorghum (*Sorghum bicolor* L.) (kindly provided by Tong Wei, UC Davis) was used to amplify SbCOMT (GenBank accession number ADW65743.1 */* Sb07g003860) using the oligonucleotides 5’-ggggacaagtttgtacaaaaaagcaggcttcatggggtcgacggcggag-3’ and 5’-gggaccactttgtacaagaaagctgggtcttacttgatgaactcgatggcccagg-3’ (Gateway sites underlined) for cloning into the Gateway pDONR221 entry vector by BP recombination (Life Technologies, Foster City, CA).

### Heterologous expression, purification and activity of SbCOMT

The pDONR221-SbCOMT entry vector was LR recombined with the pDEST17 bacterial expression vector, which introduces an N-terminal 6× His tag (Life Technologies, Foster City, CA). All vectors can be found through the Inventory of Composable Elements (ICE) at https://acs-registry.jbei.org/. Rosetta 2 (DE3) *E*. *coli* (EMD Milipore, Billerica, MA) was used for protein expression. A single bacterial colony, grown on Luria-Bertani agar containing 100 µg/mL carbenicillin and 30 µg/mL chloramphenicol was used to inoculate a 5-mL liquid culture supplemented with the same antibiotic concentrations and grown overnight at 37°C. The overnight culture was used to inoculate a 0.5-L Luria-Bertani culture at an OD_600_ = 0.05 containing the same antibiotic concentrations and grown at 37°C until it reaches an OD_600_ = 0.8–1.0. Expression was induced by the addition of 0.5 mM isopropyl-β-D-1-thiogalactopyranoside (IPTG), and the culture was transferred at 20°C and grown for 24 h. The recombinant protein was affinity purified using a HIS-Select HF Nickel Affinity Gel (Sigma-Aldrich, St. Louis, MO) according to the manufacturer’s instructions and buffer-exchanged with 50 mM Tris buffer pH 7.5 using Amicon Ultra centrifugal filters (10,000 Da MW cutoff regenerated cellulose membrane; EMD Millipore, Billerica, MA). Purity and integrity were verified by SDS-PAGE, and the recombinant protein was stored at −80°C in 50 mM Tris buffer pH 7.5, containing 10% (v/v) glycerol.

*In vitro* assays were performed at 30°C for 1 min in 50-μL reactions containing 50 mM Tris buffer pH 7.5, 1 mM DTT, 135 μM S-adenosylmethionine (BioVision Inc., Milpitas, CA), 100 ng of recombinant SbCOMT protein and 25 μM of luteolin (Ark Pharm Inc., Arlington Heights, IL), selgin, or tricetin (BroadPharm, Inc., San Diego, CA). All reactions were terminated by boiling 2 min and addition of 50% (v/v) methanol-water (50 μL) prior HPLC-ESI-TOF MS analysis performed without subsequent purification of the reaction products.

### Selgin synthesis

Selgin was synthesized as previously described [27]. Purity and integrity of the compound was validated by NMR and HPLC-ESI-TOF MS analyses (Figure A in S1 File). The NMR spectrum was recorded on a Bruker AV-600.

### Thioacidolysis

The release of tricin from cellulolytic lignin (5 mg) was conducted using the thioacidolysis procedure described in [4].

### Metabolite analyses

Metabolites were analyzed using HPLC-ESI-TOF MS as previously described [28]. Briefly, their separation was conducted on a HPX-87H column with 8% cross-linkage (150-mm length, 7.8-mm inside diameter, and 9-μm particle size; Bio-Rad, Richmond, CA) using an Agilent Technologies 1100 Series HPLC system. Metabolites were eluted isocratically with a mobile-phase composition of 0.1% formic acid in water at a flow rate of 0.5 ml/min. Drying and nebulizing gases were set to 13 liters/min and 30 lb/in^2^, respectively, and a drying-gas temperature of 330°C was used throughout. ESI was conducted in the negative ion mode and using a capillary voltage of −3,500 V. Luteolin, chrysoeriol (ChromaDex, Inc., Irvine, CA), tricin (ChromaDex, Inc., Irvine, CA), and selgin were quantified via 8-point calibration curves of authentic standard compounds for which the *R*^2^ coefficients were ≥ 0.99. Stock solutions of metabolites used for enzymatic assays and standard curves were quantified spectrophometrically using published molar absorption coefficients: S-adenosylmethionine (ε = 15,400 L.mol^-1^.cm^-1^ at 254 nm) [29], luteolin (ε = 14,790 L.mol^-1^.cm^-1^ at 350 nm) [30], chrysoeriol (ε = 15,400 L.mol^-1^.cm^-1^ at 347 nm) [30], and tricin (ε = 41,000 L.mol^-1^.cm^-1^ at 349 nm) [31].

## Results and discussion

### *Bmr12* sorghum biomass has reduced methanol-extractable tricin

**Fig 2:**
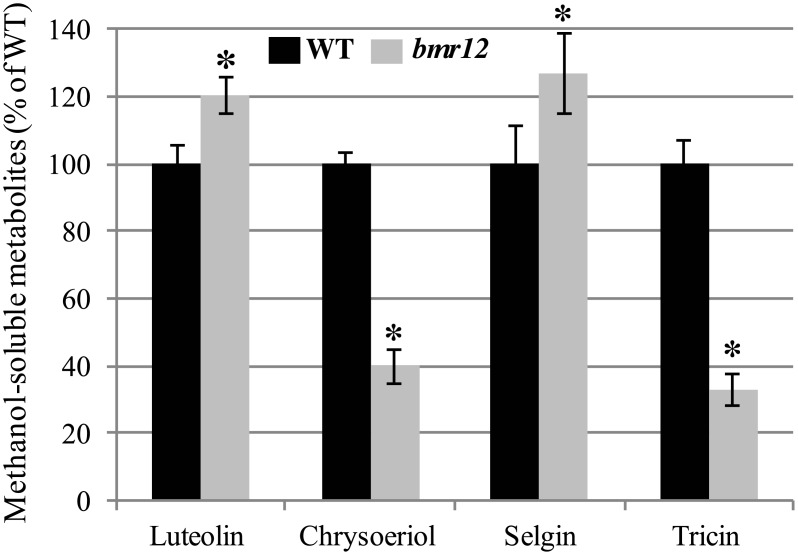
Quantification of methanol-soluble luteolin, chrysoeriol, selgin, and tricin extracted from the biomass of wild-type (WT) and *bmr12* sorghum lines. Values in *bmr12* are expressed as a percentage of the values measured in wild-type extracts which correspond to 317 ± 4 µg/g dry weight (DW) for luteolin, 7.8 ± 0.0 µg/g DW for chrysoeriol, 2.0 ± 0.2 µg/g DW for selgin, and 274 ± 3 µg/g DW for tricin. Error bars represent the standard deviation from five experimental replicates. Asterisks indicate significant differences from the wild-type using the unpaired Student’s t-test (*P < 0.05).

Methanol-soluble metabolites were extracted from total biomass of wild-type and *bmr12* plants for the quantification of tricin and its biosynthetic precursors. Tricin and chrysoeriol amounts are reduced by more than 60% in the *bmr12* mutant compared to wild-type plants, whereas luteolin and selgin contents are increased by 20% and 22%, respectively (Fig 2). Tricetin was not detected in wild-type and *bmr12* plant extracts. These results suggest a role for SbCOMT in the biosynthesis of chrysoeriol and tricin, possibly via the methylation of luteolin.

### *Bmr12* sorghum biomass has lower levels of lignin-linked tricin

**Fig 3:**
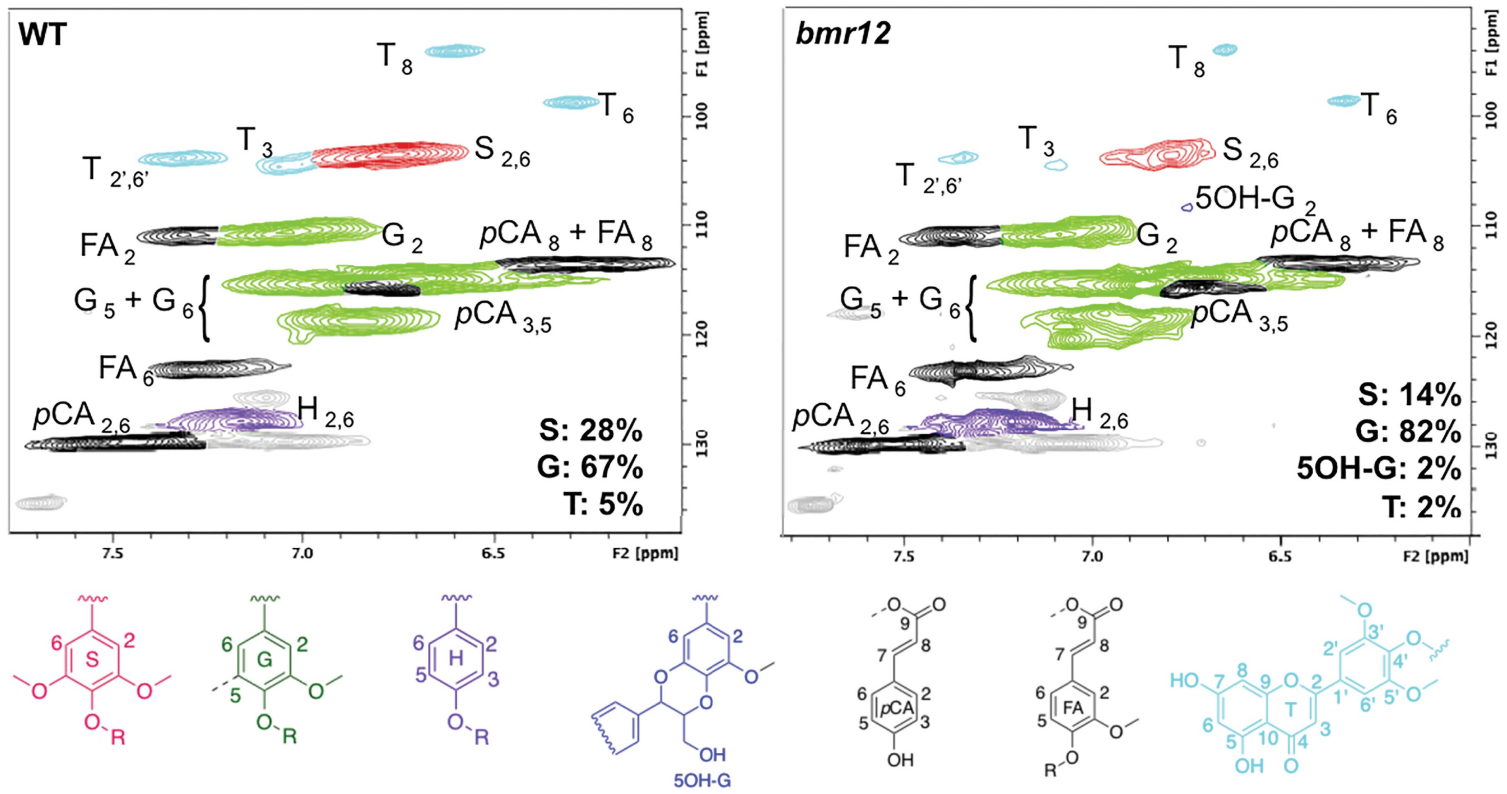
Lignin monomeric composition in wild-type (WT) and *bmr12* sorghum biomass. For each genotype, cellulolytic lignin was isolated and analyzed by 2D ^13^C–^1^H HSQC NMR spectroscopy. Regions of partial short-range ^13^C–^1^H HSQC spectra are shown. Lignin monomer ratios including tricin (T) are provided on the figures. S: syringyl, G: guaiacyl, 5OH-G: 5-hydroxyguaiacyl, H: *p*-hydroxyphenyl, *p*CA: *p*-coumarate, FA: ferulate.

Cellulolytic lignin isolated from wild-type and *bmr12* sorghum plant material was analyzed by 2D ^13^C–^1^H heteronuclear single quantum coherence (HSQC) NMR spectroscopy to determine the relative abundance of G, S, and tricin units incorporated in lignin (Fig 3). We observed in the lignin of the *bmr12* plants a 50% reduction of S units and the presence of 5-hydroxyguaiacyl (5OH-G) units resulting from the incorporation of 5-hydroxyconiferyl alcohol. In addition, benzodioxane structures, which are typically formed during β–*O*–4 coupling of a monolignol with a 5OH-G unit, were detected only in the case of *bmr12* (Figure B in S1 File). In accordance with previously published data, these observations are consistent with a reduction of SbCOMT activity, which not only impacts the synthesis of sinapaldehyde and S lignin units, but also results in the accumulation of 5-hydroxyconiferaldehyde and 5OH-G lignin units [18]. Moreover, we report here that the relative amount of tricin in the lignin of *bmr12* plants (~2%) is lower than that found in the lignin of wild-type plants (~5%) (Fig 3).

**Fig 4:**
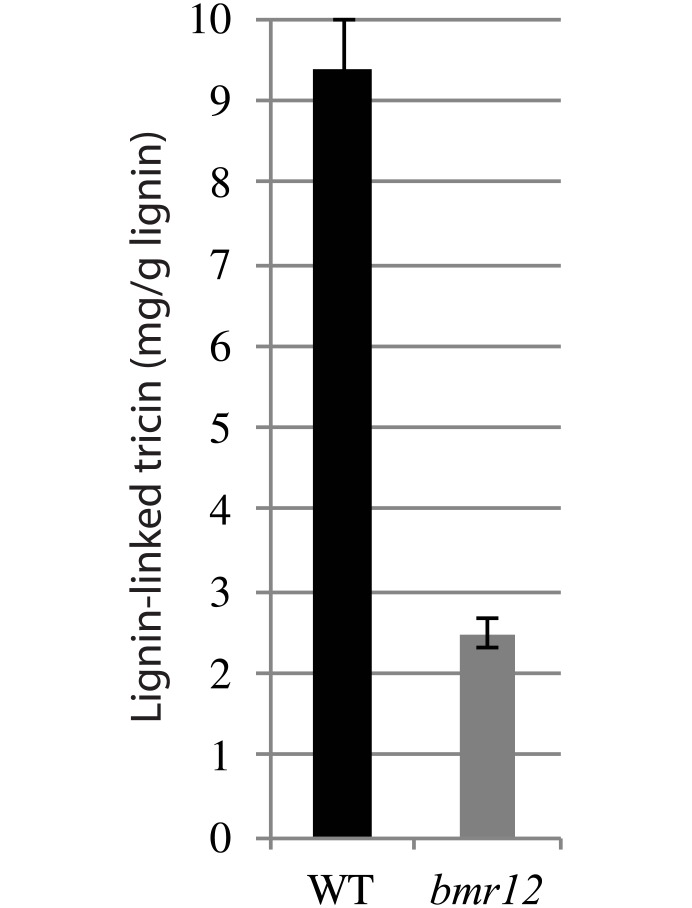
Amount of tricin in cellulolytic lignin purified from wild-type (WT) and *bmr12* sorghum lines. Tricin was released from lignin using the thioacidolysis procedure and subsequently quantified by HPLC-ESI-TOF MS. Error bars represent the standard deviation from three experimental replicates. Asterisks indicate a significant difference from the wild-type using the unpaired Student’s t-test (*P < 0.05).

To support this observation, we quantified the absolute amount of tricin incorporated in the lignin of wild-type and *bmr12* using thioacidolysis. The results showed that the lignin of wild-type plants contained 9.4 mg/g of tricin, which is consistent with previously published values obtained with this method [4], whereas the lignin of *bmr12* plants contained only 2.5 mg/g of tricin (Fig 4). These data imply that, in addition to its role in the synthesis of S-lignin units, SbCOMT is involved in the synthesis of tricin-lignin.

### SbCOMT (Bmr12) methylates luteolin, selgin, and tricetin

**Fig 5:**
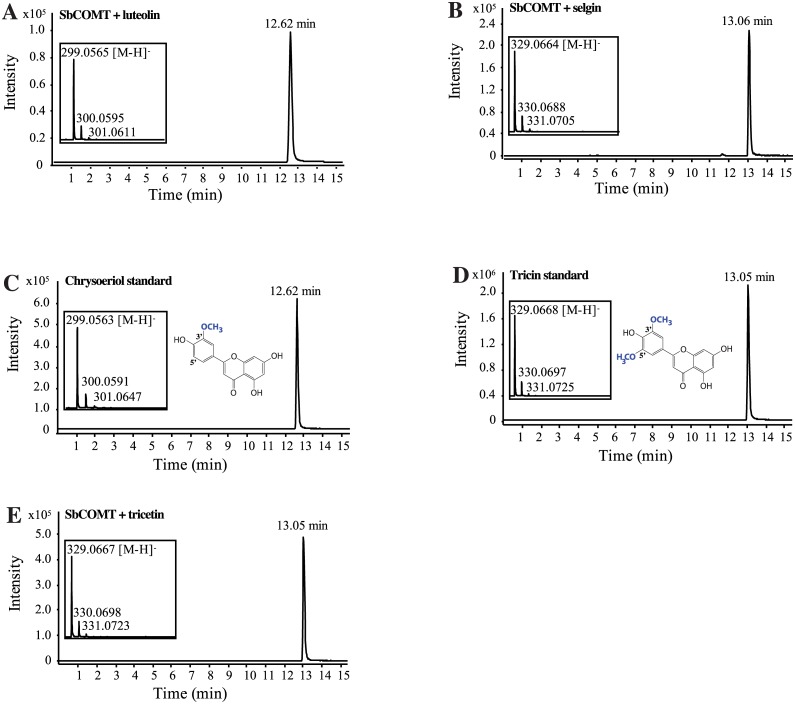
Enzymatic activity of SbCOMT (Bmr12). **(A)** Representative HPLC-ESI-TOF MS chromatogram of the chrysoeriol reaction product. Purified his-tagged SbCOMT was incubated with S-adenosylmethionine (SAM) and luteolin. **(B)** Representative HPLC-ESI-TOF MS chromatogram of the tricin reaction product. SbCOMT was incubated with SAM and selgin. **(C)** HPLC-ESI-TOF MS elution profile of a chrysoeriol standard. **(D)** HPLC-ESI-TOF MS elution profile of a tricin standard. **(E)** Representative HPLC-ESI-TOF MS chromatogram of the tricin reaction product. SbCOMT was incubated with SAM and tricetin.

Recombinant his-tagged SbCOMT was produced in *E*. *coli* and purified for biochemical characterization to assess its role in tricin biosynthesis (Figure C in S1 File). Using S-adenosylmethionine as a methyl donor, incubations of recombinant SbCOMT with luteolin or selgin (custom synthesis) resulted in the synthesis of chrysoeriol and tricin, respectively (Fig 5A and 5B), by comparison with standard compounds (Fig 5C and 5D). None of these products was observed when the reactions were carried out with a pre-boiled enzyme preparation. These results indicate that SbCOMT is able to 3’-*O*-methylate luteolin and 5’-*O*-methylates selgin. The capacity of SbCOMT to perform 3-*O*-methylation has been previously reported using caffeic acid as a substrate [17]. Finally, we observed that incubation of SbCOMT with tricetin results in the synthesis of tricin (Fig 5E), which indicates that SbCOMT 5’- and 3’-*O*-methylates this substrate.

## Conclusion

To conclude, we demonstrated in sorghum that the *O*-methyltransferase SbCOMT (Bmr12) involved in the synthesis of S lignin units also participates in the biosynthesis of the flavone tricin. Based on these results, chrysoeriol is a probable route for tricin synthesis in sorghum and the chrysoeriol 5’-hydroxylase involved in this route remains to be identified. Although our data cannot exclude the existence of a route via tricetin, to the best of our knowledge, tricetin has never been detected in sorghum. Lignin polymers incorporate tricin in several monocot species, including sorghum, and the sorghum *bmr12* mutant exhibits lower levels of lignin-linked tricin in addition to a significant reduction of S units. These observations raise questions as to whether the contribution of COMT in the synthesis of both lignin monomers, tricin and sinapyl alcohol, is specific to certain species such as sorghum and maize or whether it represents a more general mechanism. For example, it would be interesting to determine the amount of tricin in lignins from other plant species in which COMT activity is reduced such as rice, *Brachypodium*, sugarcane, alfalfa, switchgrass, and ryegrass (Figure D in S1 File) [14,32–38].

## Supporting information

S1 File Supplemental figures. **Figure A. Purity and integrity of the synthesized selgin. (A)** ^1^H NMR spectrum of synthet ic selgin. Chemical shifts (in ppm) were assigned according to the signal of the internal standard CD_3_OD (d = 3.31 ppm). **(B)** HPLC-ESI-TOF MS analysis of selgin.
**Figure B. Detection of benzodioxane substructures in lignin from *bmr12* sorghum biomass.** For each genotype, cellulolytic lignin was isolated and analyzed by 2D ^13^C–^1^H HSQC NMR spectroscopy. Regions of partial short-range ^13^C–^1^H HSQC spectra (aliphatic region) displaying the major lignin interunit structures are shown: A = β–ether (β–*O*–4´), B = phenylcoumaran (β–5´), and H = benzodioxane.
**Figure C. SDS-PAGE of purified recombinant his-tagged SbCOMT (1 µg) stained with Coomassie Brilliant Blue.** Approximate size is 42.3 kDa. The sizes of markers are indicated (kDa).
**Figure D. Phylogenetic analysis of selected *O*-methyltransferases from plant species that produce tricin.** Accession numbers are: *Sorghum bicolor* (SbCOMT, ADW65743.1), *Saccharum officinarum* (SoOMT, O82054.1), *Zea mays* (ZmCOMT, Q06509.1), *Panicum virgatum* (PvCOMT, ADX98508.1), *Oryza sativa* (OsCOMT1, XP_015650053.1), *Brachypodium dystachion* (BdCOMT6, XP_003573470.1), *Lolium perenne* (LpOMT1, AAD10253.1), *Triticum aestivum* (TaCOMT1, Q84N28.1), *Hordeum vulgare* (HvOMT, ABQ58825.1), *Triticum aestivum* (TaOMT2, Q38J50.1), *Medicago sativa* (MsCOMT, P28002.1).
(PPTX)

## References

[1] Boerjan W, Ralph J, Baucher M. Lignin biosynthesis. Annu Rev Plant Biol 2003;54:519–546. 10.1146/annurev.arplant.54.031902.134938 14503002

[2] Jouanin L, Goujon T, de Nadaï V, Martin MT, Mila I, Vallet C, et al Lignification in transgenic poplars with extremely reduced caffeic acid O-methyltransferase activity. Plant Physiol 2000;123:1363–1374. 10938354 10.1104/pp.123.4.1363 PMC59094

[3] Osakabe K, Tsao CC, Li L, Popko JL, Umezawa T, Carraway DT, et al Coniferyl aldehyde 5-hydroxylation and methylation direct syringyl lignin biosynthesis in angiosperms. Proc Natl Acad Sci U S A 1999;96:8955–8960. 10430877 10.1073/pnas.96.16.8955 PMC17714

[4] Lan W, Rencoret J, Lu F, Karlen SD, Smith BG, Harris PJ, et al Tricin-Lignins: occurrence and quantitation of tricin in relation to phylogeny. Plant J 2016;88:1046–1057. 10.1111/tpj.13315 27553717

[5] Lan W, Lu F, Regner M, Zhu Y, Rencoret J, Ralph SA, et al Tricin, a flavonoid monomer in monocot lignification. Plant Physiol 2015;167:1284–1295. 10.1104/pp.114.253757 25667313 PMC4378158

[6] Lan W, Morreel K, Lu F, Rencoret J, Carlos Del Río J, Voorend W, et al Maize tricin-oligolignol metabolites and their implications for monocot lignification. Plant Physiol 2016;171:810–820. 10.1104/pp.16.02012 27208246 PMC4902589

[7] Eloy N, Voorend W, Lan W, Saleme ML, Cesarino I, Vanholme R, et al Silencing chalcone synthase impedes the incorporation of tricin in lignin and increases lignin content. Plant Physiol 2017;173:998–1016. 10.1104/pp.16.01108 27940492 PMC5291018

[8] Lam PY, Liu H, Lo C. Completion of tricin biosynthesis pathway in rice: cytochrome P450 75B4 is a unique chrysoeriol 5'-hydroxylase. Plant Physiol 2015;168:1527–1536. 10.1104/pp.15.00566 26082402 PMC4528758

[9] Kim BG, Lee Y, Hur HG, Lim Y, Ahn J-H. Flavonoid 3'-O-methyltransferase from rice: cDNA cloning, characterization and functional expression. Phytochemistry 2006;67:387–394. 10.1016/j.phytochem.2005.11.022 16412485

[10] Lin F, Yamano G, Hasegawa M, Anzai H, Kawasaki S, Kodama O. Cloning and functional analysis of caffeic acid 3-*O*-methyltransferase from rice (*Oryza sativa*). J Pestic Sci 2006;31:47–53.

[11] Zhou J-M, Fukushi Y, Wollenweber E, Ibrahim RK. Characterization of two O-methyltransferases-like genes in barley and maize. Pharm Biol 2008;46:26–34.

[12] Zhou J-M, Fukushi Y, Wang X-F, Ibrahim RK. Characterization of a novel flavone *O*-methyltransferase gene in rice. Nat Prod Commun 2006;1:981–984.

[13] Fornalé S, Rencoret J, García-Calvo L, Encina A, Rigau J, Gutiérrez A, et al Changes In cell wall polymers and degradability in maize mutants lacking 3'- and 5'-O-methyltransferases involved in lignin biosynthesis. Plant Cell Physiol 2017;58:240–255. 10.1093/pcp/pcw198 28013276

[14] Koshiba T, Hirose N, Mukai M, Yamamura M, Hattori T, Suzuki S, et al (2013). Characterization of 5-hydroxyconiferaldehyde *O*-methyltransferase in *Oryza sativa*. Plant Biotechnol 2013;30:157–167.

[15] Piquemal J, Chamayou S, Nadaud I, Beckert M, Barrière Y, Mila I, et al Down-regulation of caffeic acid *O*-methyltransferase in maize revisited using a transgenic approach. Plant Physiol 2002;130:1675–1685. 10.1104/pp.012237 12481050 PMC166682

[16] Bout S, Vermerris W. A candidate-gene approach to clone the sorghum Brown midrib gene encoding caffeic acid *O*-methyltransferase. Mol Genet Genomics 2003;269:205–214. 10.1007/s00438-003-0824-4 12756532

[17] Green AR, Lewis KM, Barr JT, Jones JP, Lu F, Ralph J, et al Determination of the structure and catalytic mechanism of *sorghum bicolor* caffeic acid *O*-methyltransferase and the structural impact of three *brown midrib12* mutations. Plant Physiol 2014;165:1440–1456. 10.1104/pp.114.241729 24948836 PMC4119030

[18] Palmer NA, Sattler SE, Saathoff AJ, Funnell D, Pedersen JF, Sarath G. Genetic background impacts soluble and cell wall-bound aromatics in *brown midrib* mutants of sorghum. Planta 2008;229:115–127. 10.1007/s00425-008-0814-1 18795321

[19] Sattler SE, Funnell-Harris DL, Pedersen JF. Efficacy of singular and stacked *brown midrib 6* and *12* in the modification of lignocellulose and grain chemistry. J Agric Food Chem 2010;58:3611*–*3616. 10.1021/jf903784j 20175527

[20] Eudes A, George A, Mukerjee P, Kim JS, Pollet B, Benke PI, et al Biosynthesis and incorporation of side-chain-truncated lignin monomers to reduce lignin polymerization and enhance saccharification. Plant Biotechnol J 2012;10:609–620. 10.1111/j.1467-7652.2012.00692.x 22458713

[21] Heikkinen S, Toikka MM, Karhunen PT, Kilpeläinen IA. Quantitative 2D HSQC (Q-HSQC) via suppression of J-dependence of polarization transfer in NMR spectroscopy: application to wood lignin. J Am Chem Soc 2003;125:4362–4367. 10.1021/ja029035k 12670260

[22] del Rio JC, Rencoret J, Prinsen P, Martinez AT, Ralph J, Gutierrez A. Structural characterization of wheat straw lignin as revealed by analytical pyrolysis, 2D-NMR, and reductive cleavage methods. J Agric Food Chem 2012;60:5922–5935. 10.1021/jf301002n 22607527

[23] Kim H, Ralph J. Solution-state 2D NMR of ball-milled plant cell wall gels in DMSO-d(6)/pyridine-d(5). Org Biomol Chem 2010;8:576–591. 10.1039/b916070a 20090974 PMC4070321

[24] Vanholme R, Ralph J, Akiyama T, Lu F, Pazo JR, Kim H, et al Engineering traditional monolignols out of lignin by concomitant up-regulation of F5H1 and down-regulation of COMT in Arabidopsis. Plant J 2010;64:885*–*897. 10.1111/j.1365-313X.2010.04353.x 20822504

[25] Yelle DJ, Ralph J, Frihart CR. Characterization of nonderivatized plant cell walls using high-resolution solution-state NMR spectroscopy. Magn Reson Chem 2008;46:508–517. 10.1002/mrc.2201 18383438 PMC5826555

[26] Mansfield SD, Kim H, Lu F, Ralph J. Whole plant cell wall characterization using solution-state 2D NMR. Nat Protoc 2012;1579–1589. 10.1038/nprot.2012.064 22864199

[27] Feng J-P, Wang X-L, Cao X-P. The first total synthesis of the (±)-palstatin. Chin J Chem 2006;24:215–218.

[28] Eudes A, Juminaga D, Baidoo EE, Collins FW, Keasling JD, Loqué D. Production of hydroxycinnamoyl anthranilates from glucose in *Escherichia coli*. Microb Cell Fact 2013;12:62 10.1186/1475-2859-12-62 23806124 PMC3716870

[29] Huber TD, Wang F, Singh S, Johnson BR, Zhang J, Sunkara M, et al Functional AdoMet isosteres resistant to classical AdoMet degradation pathways. ACS Chem Biol 2016;11:2484–2491. 10.1021/acschembio.6b00348 27351335 PMC5026608

[30] Hartwig UA, Maxwell CA, Joseph CM, Phillips DA. Chrysoeriol and luteolin released from alfalfa seeds induce nod genes in *Rhizobium meliloti*. Plant Physiol 1990;92:116–122. 16667231 10.1104/pp.92.1.116 PMC1062256

[31] Matsuta T, Sakagami H, Satoh K, Kanamoto T, Terakubo S, Nakashima H, et al Biological activity of luteolin glycosides and tricin from Sasa senanensis Rehder. In Vivo 2011;25:757–762. 21753130

[32] Fu C, Mielenz JR, Xiao X, Ge Y, Hamilton CY, Rodriguez M et al Genetic manipulation of lignin reduces recalcitrance and improves ethanol production from switchgrass. Proc Natl Acad Sci U S A 2011;108:3803–3808. 10.1073/pnas.1100310108 21321194 PMC3048149

[33] Guo D, Chen F, Inoue K, Blount JW, Dixon RA. Downregulation of caffeic acid 3-*O*-methyltransferase and caffeoyl CoA 3-*O*-methyltransferase in transgenic alfalfa: Impacts on lignin structure and implications for the biosynthesis of G and S lignin. Plant Cell 2011;13:73–88. 10.1105/tpc.13.1.73 PMC102215 11158530

[34] Ho-Yue-Kuang S, Alvarado C, Antelme S, Bouchet B, Cézard L, Le Bris P, et al Mutation in *Brachypodium* caffeic acid *O*-methyltransferase 6 alters stem and grain lignins and improves straw saccharification without deteriorating grain quality. J Exp Bot 2016;67:227–237. 10.1093/jxb/erv446 26433202 PMC4682429

[35] Jung JH, Fouad WM, Vermerris W, Gallo M, Altpeter F. RNAi suppression of lignin biosynthesis in sugarcane reduces recalcitrance for biofuel production from lignocellulosic biomass. Plant Biotechnol J 2012;10:1067–1076. 10.1111/j.1467-7652.2012.00734.x 22924974

[36] Tu Y, Rochfort S, Liu Z, Ran Y, Griffith M, Badenhorst P, et al Functional analyses of caffeic acid *O*-methyltransferase and cinnamoyl-CoA-reductase genes from perennial ryegrass (*Lolium perenne*). Plant Cell 2010;22:3357–3373. 10.1105/tpc.109.072827 20952635 PMC2990129

[37] Zhou J-M, Seo YW, Ibrahim RK. Biochemical characterization of a putative wheat caffeic acid *O*-methyltransferase. Plant Physiol Biochem 2009;47:322–326 10.1016/j.plaphy.2008.11.011 19211254

[38] Zhou J-M, Gold ND, Martin VJ, Wollenweber E, Ibrahim RK. Sequential O-methylation of tricetin by a single gene product in wheat. Biochim Biophys Acta 2006;1760:1115–1124. 10.1016/j.bbagen.2006.02.008 16730127

