# Supplementary material for: SbCOMT (Bmr12) is involved in the biosynthesis of tricin-lignin in sorghum
Source: PLoS One. 2017 Jun 8;12(6):e0178160. doi: 10.1371/journal.pone.0178160 (PMC5464547; doi:10.1371/journal.pone.0178160)
Supplement: S1 File — Figure A. Purity and integrity of the synthesized selgin. (A) 1H NMR spectrum of synthet ic selgin. Chemical shifts (in ppm) were assigned according to the signal of the internal standard CD3OD (d = 3.31 ppm). (B) HPLC-ESI-TOF MS analysis of selgin. Figure B. Detection of benzodioxane substructures in lignin from bmr12 sorghum biomass. For each genotype, cellulolytic lignin was isolated and analyzed by 2D 13C–1H HSQC NMR spectroscopy. Regions of partial short-range 13C–1H HSQC spectra (aliphatic region) displaying the major lignin interunit structures are shown: A = β–ether (β–O–4´), B = phenylcoumaran (β–5´), and H = benzodioxane. Figure C. SDS-PAGE of purified recombinant his-tagged SbCOMT (1 µg) stained with Coomassie Brilliant Blue. Approximate size is 42.3 kDa. The sizes of markers are indicated (kDa). Figure D. Phylogenetic analysis of selected O-methyltransferases from plant species that produce tricin. Accession numbers are: Sorghum bicolor (SbCOMT, ADW65743.1), Saccharum officinarum (SoOMT, O82054.1), Zea mays (ZmCOMT, Q06509.1), Panicum virgatum (PvCOMT, ADX98508.1), Oryza sativa (OsCOMT1, XP_015650053.1), Brachypodium dystachion (BdCOMT6, XP_003573470.1), Lolium perenne (LpOMT1, AAD10253.1), Triticum aestivum (TaCOMT1, Q84N28.1), Hordeum vulgare (HvOMT, ABQ58825.1), Triticum aestivum (TaOMT2, Q38J50.1), Medicago sativa (MsCOMT, P28002.1). (PPTX) [file pone.0178160.s001.pptx]

## Slide 1
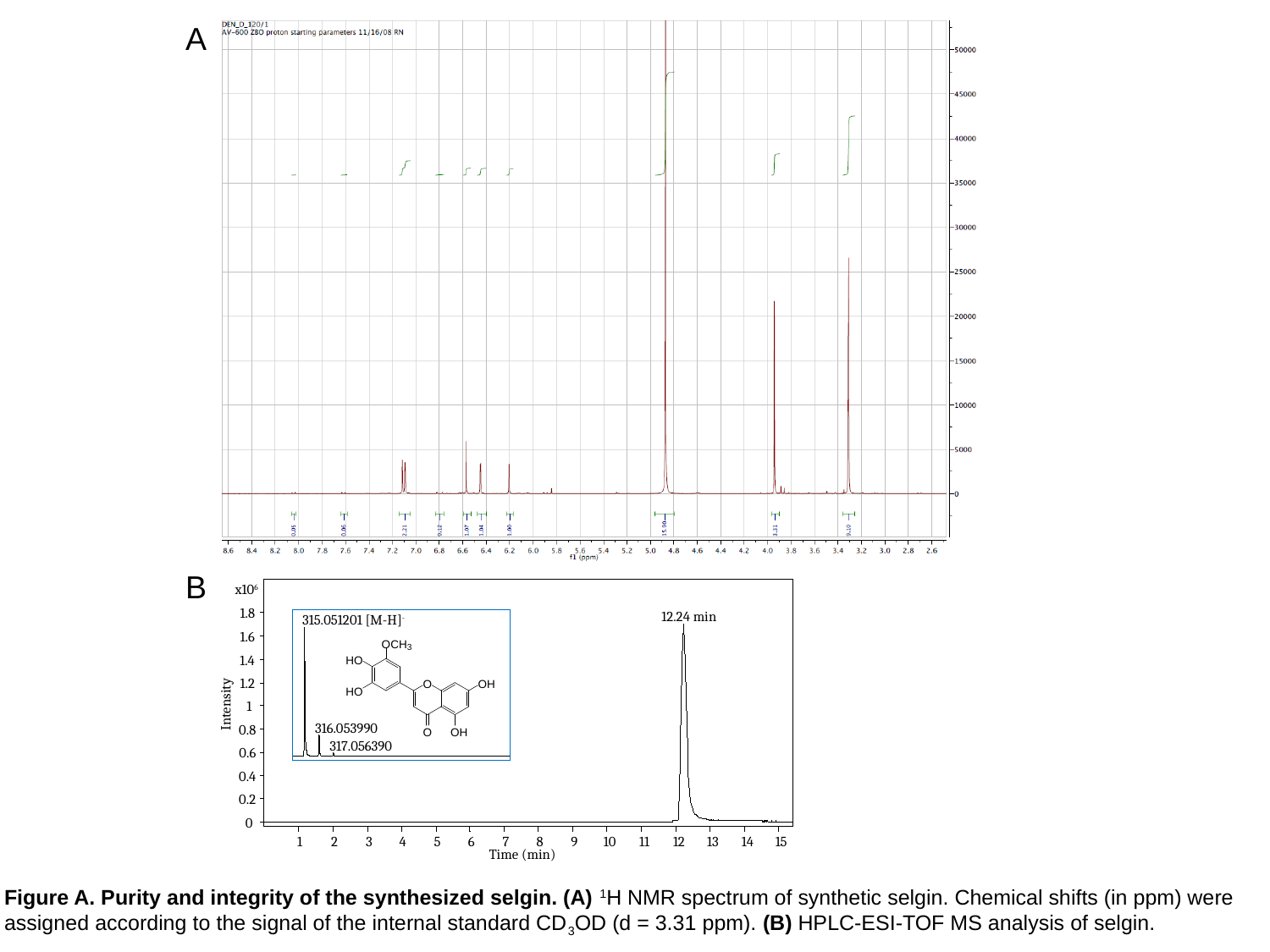

A
B
x106
1.8
12.24 min
315.051201 [M-H]-
1.6
1.4
1.2
Intensity
1
316.053990
0.8
317.056390
0.6
0.4
0.2
0
1
2
3
4
5
6
7
8
9
10
11
12
13
14
15
Time (min)
Figure A. Purity and integrity of the synthesized selgin. (A) 1H NMR spectrum of synthetic selgin. Chemical shifts (in ppm) were assigned according to the signal of the internal standard CD3OD (d = 3.31 ppm). (B) HPLC-ESI-TOF MS analysis of selgin.

## Slide 2
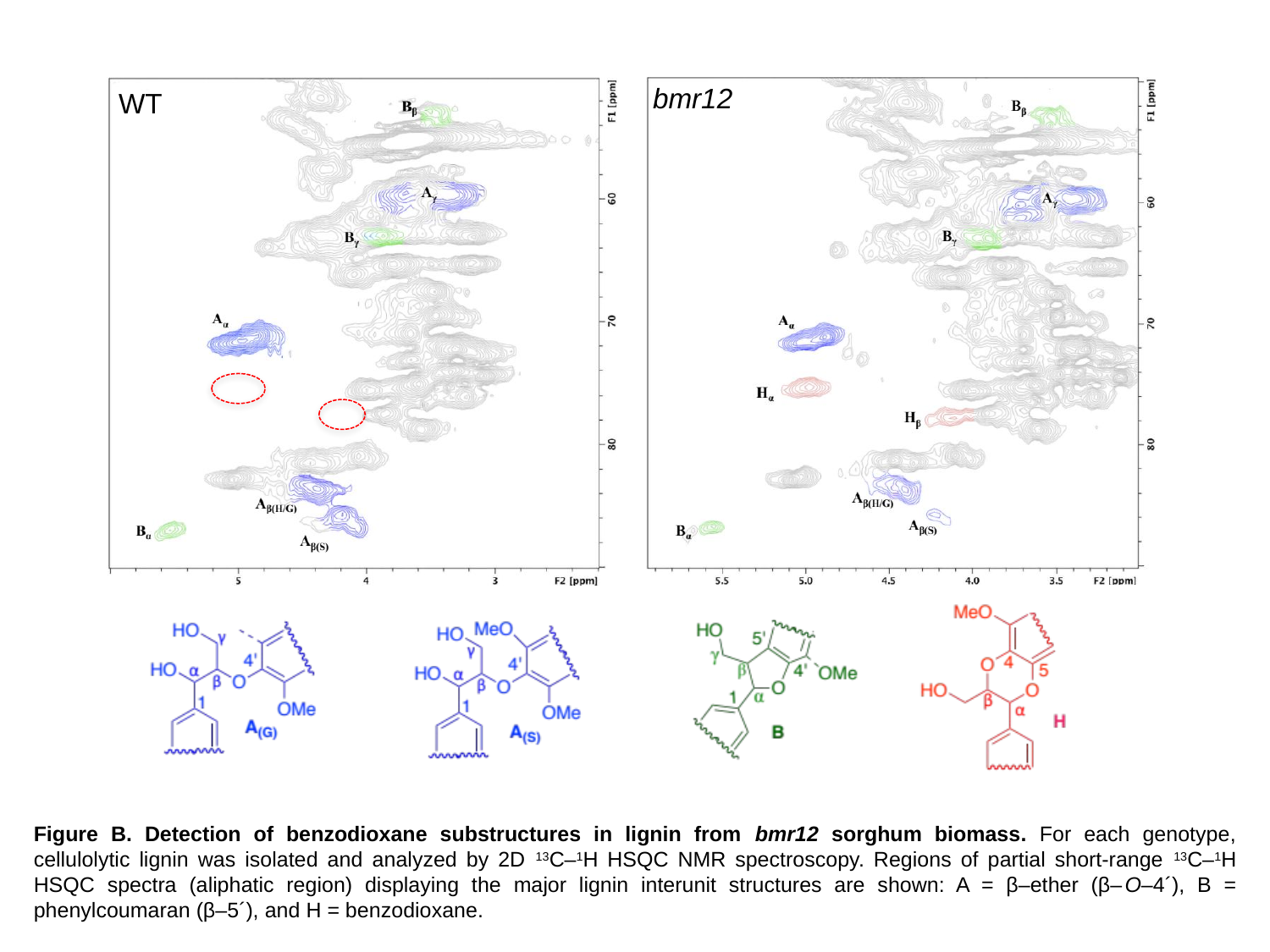

bmr12
WT
Figure B. Detection of benzodioxane substructures in lignin from bmr12 sorghum biomass. For each genotype, cellulolytic lignin was isolated and analyzed by 2D 13C–1H HSQC NMR spectroscopy. Regions of partial short-range 13C–1H HSQC spectra (aliphatic region) displaying the major lignin interunit structures are shown: A = β–ether (β–O–4´), B = phenylcoumaran (β–5´), and H = benzodioxane.

## Slide 3
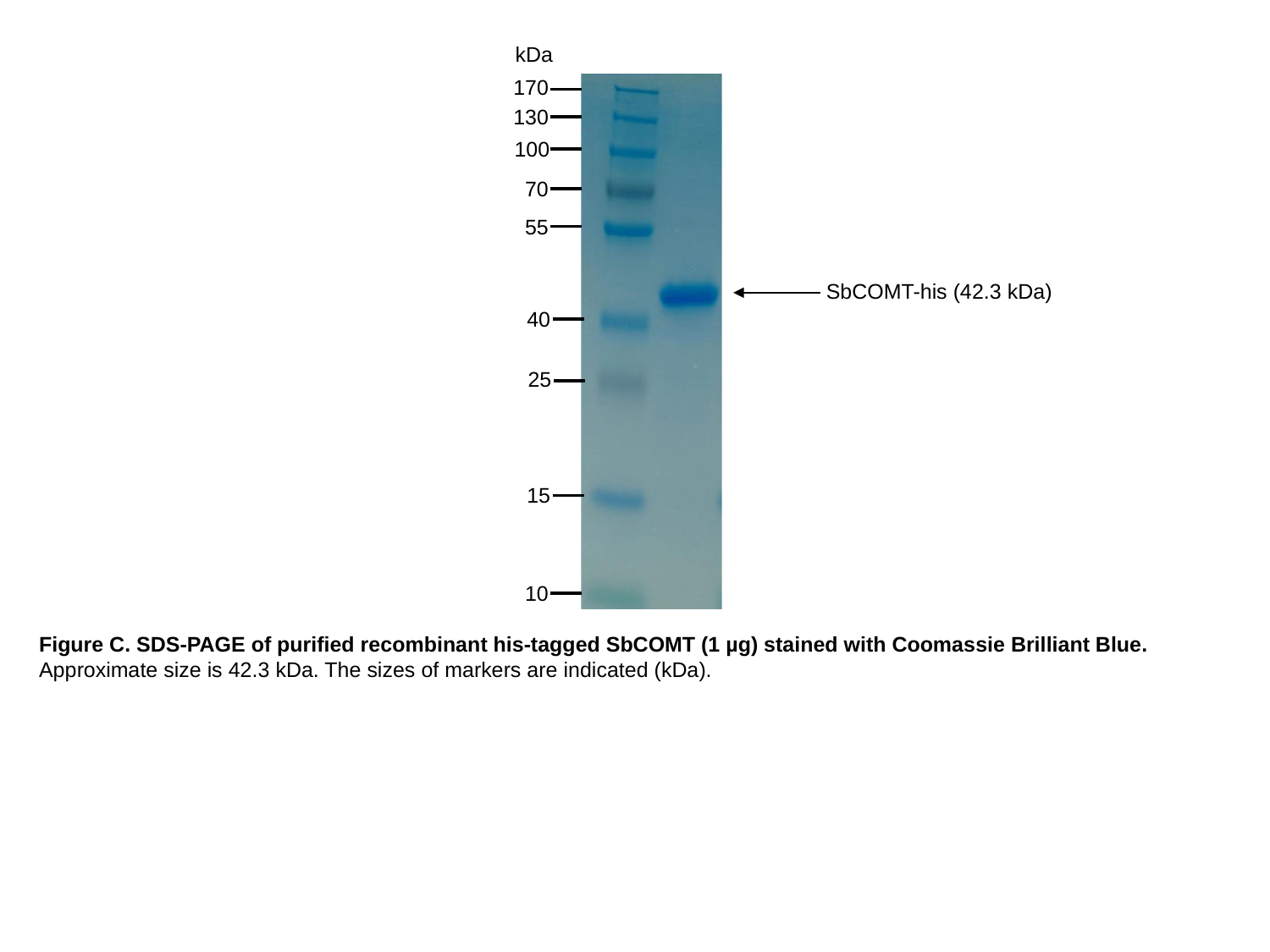

kDa
170
130
100
70
55
SbCOMT-his (42.3 kDa)
40
25
15
10
Figure C. SDS-PAGE of purified recombinant his-tagged SbCOMT (1 µg) stained with Coomassie Brilliant Blue.
Approximate size is 42.3 kDa. The sizes of markers are indicated (kDa).

## Slide 4
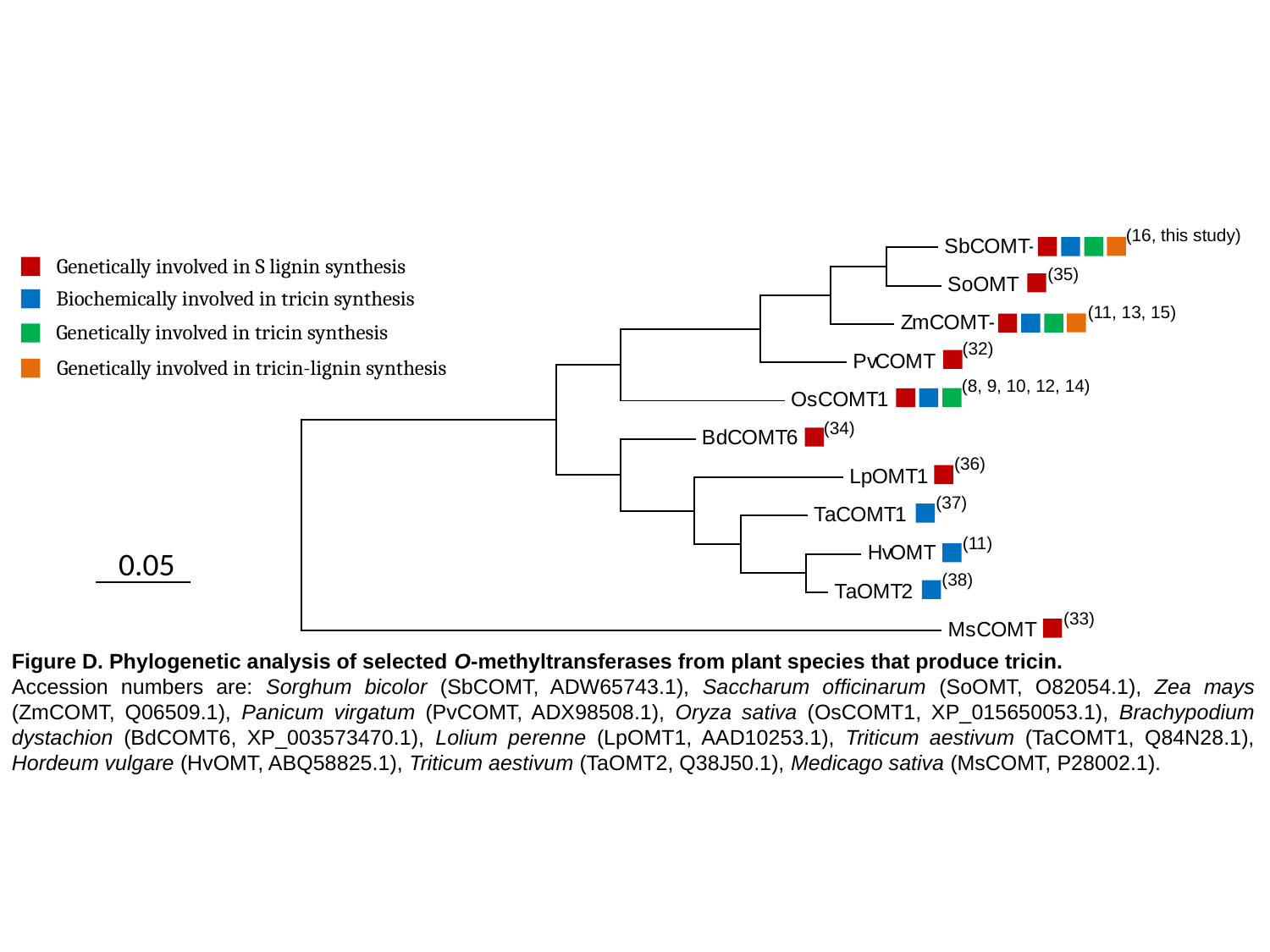

(16, this study)
Genetically involved in S lignin synthesis
(35)
Biochemically involved in tricin synthesis
(11, 13, 15)
Genetically involved in tricin synthesis
(32)
Genetically involved in tricin-lignin synthesis
(8, 9, 10, 12, 14)
(34)
(36)
(37)
(11)
0.05
(38)
(33)
Figure D. Phylogenetic analysis of selected O-methyltransferases from plant species that produce tricin.
Accession numbers are: Sorghum bicolor (SbCOMT, ADW65743.1), Saccharum officinarum (SoOMT, O82054.1), Zea mays (ZmCOMT, Q06509.1), Panicum virgatum (PvCOMT, ADX98508.1), Oryza sativa (OsCOMT1, XP_015650053.1), Brachypodium dystachion (BdCOMT6, XP_003573470.1), Lolium perenne (LpOMT1, AAD10253.1), Triticum aestivum (TaCOMT1, Q84N28.1), Hordeum vulgare (HvOMT, ABQ58825.1), Triticum aestivum (TaOMT2, Q38J50.1), Medicago sativa (MsCOMT, P28002.1).
